# Oxidative Stress-Related Mechanisms in Melanoma and in the Acquired Resistance to Targeted Therapies

**DOI:** 10.3390/antiox10121942

**Published:** 2021-12-03

**Authors:** Stefania Pizzimenti, Simone Ribero, Marie Angele Cucci, Margherita Grattarola, Chiara Monge, Chiara Dianzani, Giuseppina Barrera, Giuliana Muzio

**Affiliations:** 1 Department of Clinical and Biological Science, University of Turin, 10125 Turin, Italy; marieangele.cucci@unito.it (M.A.C.); margherita.grattarola@unito.it (M.G.); giuseppina.barrera@unito.it (G.B.); giuliana.muzio@unito.it (G.M.); 2 Department of Medical Sciences, Section of Dermatology, University of Turin, 10126 Turin, Italy; simone.ribero@unito.it; 3 Department of Scienza e Tecnologia del Farmaco, University of Turin, 10125 Turin, Italy; chiara.monge@unito.it (C.M.); chiara.dianzani@unito.it (C.D.)

**Keywords:** melanoma, targeted therapies, BRAFi, MEKi, resistance, oxidative stress, ROS, RNS, antioxidants, Nrf2, lipid peroxidation, ALDH

## Abstract

Melanoma is a highly aggressive cancer with the poorest prognosis, representing the deadliest form of skin cancer. Activating mutations in BRAF are the most frequent genetic alterations, present in approximately 50% of all melanoma cases. The use of specific inhibitors towards mutant BRAF variants and MEK, a downstream signaling target of BRAF in the MAPK pathway, has significantly improved progression-free and overall survival in advanced melanoma patients carrying BRAF mutations. Nevertheless, despite these improvements, resistance still develops within the first year of therapy in around 50% of patients, which is a significant problem in managing BRAF-mutated advanced melanoma. Understanding these mechanisms is one of the mainstreams of the research on BRAFi/MEKi acquired resistance. Both genetic and epigenetic mechanisms have been described. Moreover, in recent years, oxidative stress has emerged as another major force involved in all the phases of melanoma development, from initiation to progression until the onsets of the metastatic phenotype and chemoresistance, and has thus become a target for therapy. In the present review, we discuss the current knowledge on oxidative stress and its signaling in melanoma, as well as the oxidative stress-related mechanisms in the acquired resistance to targeted therapies.

## 1. Melanoma and Targeted Therapies

Melanoma, which originates from a malignant transformation of melanocytes, is the third most common malignant tumor of the skin, after the most frequent basal cell carcinoma and the squamous cell carcinoma, both of which arise from keratinocytes or their precursors. However, melanoma is the most threatening cancer with the poorest prognosis, representing the deadliest form of skin cancer.

Globally, in 2020, new melanoma cases occurred in 324,635 people and resulted in 57,043 deaths [1]. Over the past four decades, the incidence of melanoma has increased throughout the world, with the greatest incidence rates in predominantly fair-skinned populations living in New Zealand, Australia, North America, and Europe [2,3].

Metastatic melanoma (MM) is poorly responsive to conventional chemotherapeutic regimens, with an estimated 5-year survival rate of about 15% [4]. However, over the past few years, new targeted treatments and immunotherapy [5] have significantly improved the global approach toward melanoma.

For MM patients harboring the cytoplasmic serine/threonine kinase B-Raf (BRAF) wild type, current guidelines recommend the use of monoclonal antibodies targeting immune checkpoint proteins such the anti-programmed death 1 (PD-1) (pembrolizumab or nivolumab) or the cytotoxic T-lymphocyte antigen 4 (CTLA-4) (ipilimumab) in combination with an anti-PD-1 therapy [6].

Activating mutations in BRAF are the most frequent genetic alterations, present in approximately 50% of all melanoma cases [7], and more than 90% of those have an activating valine–glutamic acid substitution in codon 600 of the BRAF (V600E) [8]. Furthermore, other, less frequent missense mutations of this codon have been described in melanoma, such as V600K, V600R, and V600D [9]. The activating V600E mutation is considered a phosphomimetic substitution and brings to a constitute activation of the mitogen-activated protein kinase (MAPK) signaling pathway. V600E mutated BRAF has been found in younger patients and in more aggressive disease. BRAF inhibitors (BRAFi) such as vemurafenib and dabrafenib have been successfully employed in patients with advanced V600E/K BRAF melanoma diseases. These treatments resulted in an increase in median progression-free survival compared to dacarbazine, which was considered the most effective single chemotherapeutic agent for the treatment of advanced MM in use for more than 30 years [10].

However, following the initially enthusiastic response rates, resistance to the targeted therapy emerges with a median time to progression of 5.1–8.8 months, quite often caused by a reactivation of the MAPK pathway [11]. Thus, new therapeutic options aim to simultaneously target both BRAF and its downstream effector, mitogen-activated protein kinase (MEK), to overcome one of the most important genetic mechanisms of escape. Indeed, when comparing BRAFi/MEKi combination therapy with BRAFi alone, increases in progression-free survival (PFS) and the overall survival (OS) have been proven [12].

Patients treated with the dabrafenib (BRAFi)–trametinib (MEKi) combination showed 26.1 months OS, with a significant improvement over the 17.8 months OS observed in patients treated with vemurafenib (BRAFi) monotherapy. Median PFS in the dabrafenib–trametinib arm was 12.1 months, and that in the vemurafenib arm was 7.3 months [13]. Similar results were obtained with the dabrafenib–trametinib combination when compared with dabrafenib alone: 11 months was the median PFS for the patients in the combination therapy arm, compared to 8.8 months in the monotherapy arm; 25.1 and 18.7 months, respectively, were the median OS [14]. Quality of life and side effect analysis favored the combination over the monotherapy [13,14,15].

Furthermore, in a phase 3 randomized clinical trial (coBRIM), the combination of vemurafenib and cobimetinib (MEKi) was more effective than vemurafenib alone in BRAF-mutated patients with advanced disease. The combination showed a significantly higher response rate (RR) (68% in the combination therapy arm versus 45% in the monotherapy arm), PFS (9.9 versus 6.2 months, respectively), and 9-month survival rates (81% versus 73%, respectively) [16].

More recently, the new anti-BRAF agent encorafenib, in combination with the MEKi binimetinib, was investigated in the COLUMBUS trial. At 3-year analysis, the combination showed a median OS of 33.6 months, while vemurafenib alone showed a median OS of 16.9 months [17]; moreover, the median PFS was 14.9 months with the combination and 7.3 months with monotherapy. Nevertheless, despite these improvements, after the combined treatment, nearly 50% of patients developed resistance within the first year of the targeted therapy, representing the most challenging management problem of the disease [12].

Understanding these mechanisms is still one of the mainstreams of the research on BRAFi/MEKi acquired resistance. However, high heterogeneity among patients and within individual tumors makes difficult to fully identify them. Nevertheless, a number of genetic and epigenetic mechanisms have been described in BRAF/MEKi resistance. Genetic alterations providing resistance to BRAFi are found in the majority of resistant tumors. In melanoma patients resistant to targeted therapy, mutations leading to the reactivation of the MAPK pathway are the most representative [18]. BRAFV600 amplifications, BRAF splice site variants, and activating mutations of NRAS and MEK1/2 have been described [19].

In addition to reactivation of the MAPK pathway, in around 20% of melanoma patients developing resistance to targeted therapy, there is increased signaling in the phosphatidylinositol 3-kinase (PI3K) and protein kinase B (AKT) pathway [20]. Activating mutations of PI3K and AKT have been described as responsible for the upregulation of this signal pathway. Unfortunately, combined use of BRAFi/MEKi with PI3K/AKT inhibitors failed to show benefit in clinical trial studies [21].

Not only are MAPK and PI3K pathways upregulated in resistant melanoma cells, but so are their upstream receptor tyrosine kinases (RTKs), such as the Erb-B2 receptor tyrosine kinase 3 (ERBB3), the insulin-like growth factor (IGF)-1 receptor, the hepatocyte growth factor receptor (c-MET), the epidermal growth factor receptor (EGFR), and the platelet-derived growth factor receptor α (PDGFR α) [21,22,23,24,25,26]. Unfortunately, BRAFi treatment elicits the activation of multiple RTKs in the same tumor, and not a selective upregulation of specific receptors; this feature hinders the possibility of a therapeutic intervention aimed at a particular type of RTK [21]. Several other non-MAPK pathway dysregulations have been described in BRAFi/MEKi resistance, such as the copy number variations of cyclin D1 and cyclin-dependent kinase inhibitor p16INK4A and the inactivation of retinoblastoma (Rb) protein [27,28].

Epigenetic mechanisms can also participate in acquired BRAFi/MEKi resistance. DNA methylation pattern at individual CpG sites [29], post-translational modifications of histones, and noncoding RNA deregulation have been demonstrated to be involved [30]. These epigenetic mechanisms profoundly affect the expression of critical genes involved in favoring the growth and progression of melanoma, such as a set of transcriptional “master regulators” [31]. For instance, the upregulation of the transcription factor c-JUN participates in the acquisition of epithelial–mesenchymal transition (EMT)-like phenotypes in melanoma cells [32]. The inhibition of this transcriptional master regulator together with BRAFi [33] elicited cell death decreased a population of melanoma cells with a mesenchymal-like phenotype, which represents an early adaptive state adopted by some melanoma cells in response to BRAFi [33,34,35]. Microphthalmia-associated transcription factor (MITF), involved in melanocyte development, was found to be upregulated in BRAFi/MEKi resistance. On the contrary, other studies have demonstrated that during the acquisition of the resistance to targeted therapies, a population of melanoma cells exhibited low MITF expression [36].

In addition to these molecular mechanisms, in recent years, researchers have focused their attention on oxidative stress as a major force in eliciting genetic mutations and controlling gene expression. Indeed, redox biology, which includes reactive oxygen and nitrogen species (ROS and RNS) and antioxidants, plays a central role in tumors, including melanoma. In the present review, we discuss the current knowledge on oxidative stress and its signaling in melanoma and the oxidative stress-related mechanisms in acquired resistance to targeted therapies.

## 2. Oxidative Stress in Physiopathology and in Cancer

Oxidative stress describes an imbalance between the production of ROS/RNS in tissues and the ability, from the biological systems, to detoxify these highly reactive species or to repair the resulting damage [37,38]. ROS/RNS, which are constantly produced in living organisms, include free radicals (superoxide anion O_2_^•−^; hydroxyl radical ^•^OH; nitric oxide NO^•^; nitrogen dioxide ^•^NO_2_) and nonradicals, such as singlet oxygen (1O_2_), peroxides (hydrogen peroxide H_2_O_2_; peroxynitrite ONOO^−^), and hypochlorous acid (HOCl) [39,40].

ROS can be generated by nonenzymatic processes, such as the Fenton/Haber–Weiss reaction, and enzymatic reactions, which include the mitochondrial electron transport chain, nicotinamide adenine dinucleotide phosphate (NADPH) oxidases (NOXs), cyclooxygenases, or xanthine oxidases [40].

ROS can act as intracellular signaling molecules, participating in the homeostatic adaptation of cells to external stimuli [41,42]. During inflammation, ROS can be overproduced since they are highly toxic to pathogens [43].

In the condition of oxidative stress, ROS/RNS are produced in excess, and they are very harmful compounds. At high concentrations, they can damage cellular DNA, proteins, and lipids. In lipid membranes, ROS/RNS trigger polyunsaturated fatty acid (PUFA) oxidative degradations. This process is known as lipid peroxidation (LPO) and induces the formation of lipid radicals and other reactive intermediates (lipid radical L^•^, lipid peroxy radical L-OO^•^, lipid hydroperoxide L-OOH), as well as several highly reactive aldehydes, including 4-hydroxynonenal (HNE) and malondialdehyde (MDA), further amplifying the toxic effect of free radicals [44,45].

Prof. Mario Umberto Dianzani [46], to whom this special issue is dedicated, was the first scientist who ever described the occurrence of lipid peroxidation in relevant pathophysiological conditions, such as carbon tetrachloride-induced liver injury [47]. Together with Prof. Esterbauer [48], the first scientist who demonstrated the biological origin of HNE [49], Prof. Dianzani gave a fundamental contribution to the comprehension of the role played by HNE and other aldehydic products derived from lipid peroxidation in human diseases [50,51].

ROS/RNS and LPO products have been identified as major players in several diseases, such as diabetes, rheumatoid arthritis, stroke, cardiovascular diseases, atherosclerosis, chronic inflammatory pathologies, aging-related disorders, autoimmune diseases, neurodegenerative conditions, and cancer [39,45,52,53,54,55,56,57,58].

Several enzymes and molecules present in our tissues can protect cells from oxidative stress damage, being able to neutralize free radicals and metabolize the toxic aldehydes produced during LPO. They include both endogenous and exogenous molecules acting through enzymatic and nonenzymatic pathways. The endogenous antioxidant defense system enzymes are represented by glutathione peroxidases (GPXs), superoxide dismutases (SODs), glutathione S-transferases (GSTs), catalases (CAT), thioredoxins (TRXs), thioredoxin peroxidases (TRXPs), peroxiredoxins (PRDXs), and heme oxygenase-1 (HO-1); nonenzymatic molecules with antioxidant properties include tripeptide glutathione (GSH), proteins (i.e., ferritin, transferrin, albumin, ceruloplasmin), and several low-molecular-weight scavengers (uric acid, coenzyme Q, and lipoic acid). Plant-derived exogenous antioxidants present in fruits and vegetables, such as Vitamin C, E, and A, are also involved in free radical detoxification [59,60]. Furthermore, several protective metabolic pathways are responsible for the rapid intracellular catabolism of the toxic aldehydes produced during LPO. For instance, HNE, one of the most extensively studied products of LPO, can be metabolized by aldehyde dehydrogenases (ALDH), aldo/keto-reductase (AKR), alcohol dehydrogenase (ADH), and GST [61,62].

One of the main proteins involved in regulating antioxidant response is the transcription factor Nrf2 (NF-E2-related factor 2), which has been considered the master regulator of cytoprotective and antioxidant genes [63,64]. In physiological conditions, Nrf2 is found in the cytosol linked to its inhibitor, Keap1 (Kelch-like ECH-associated protein), leading to inactivation via ubiquitination and proteasomal degradation. Under oxidative stress, Keap1 become oxidized in its cysteine residues, undergoes a conformational change, and releases Nrf2, which translocates into the nucleus and binds to the antioxidant response element (ARE) sequences present in the promoter of genes coding for aforementioned antioxidant enzymes, such as HO-1, PRDXs, TXN, as well as genes involved in GSH synthesis, such as γ-glutamate-cysteine ligase (GCL), which catalyzes the first step in the production of GSH, and xCT, which codifies the light chain component of the system X_c_^-^. X_c_^-^ is an antiporter able to export glutamate and import cystine, which is then reduced to cysteine and used for the synthesis of GSH [65].

A mountain of evidence has suggested that redox imbalance and resulting lipid peroxidation products play an important role in cancer development, progression, metastasis, and chemoresistance. These findings, excellently presented in several reviews [56,66,67,68,69,70,71,72,73], are far from being unique. For instance, scientists have highlighted some paradoxes relating to the two faces of ROS, which are able to both promote and suppress cancer [74]. Similarly, Nrf2 and its antioxidant target genes are regarded as double-edged swords, with their anticancer and protumoral activities, depending on the stages of malignant transformation [75].

In this scenario, melanomas are not an exception. It has been demonstrated that ROS/RNS play a central role in all the phases of the disease, from the initiation to progression until the onsets of the metastatic phenotype and chemoresistance, including that to targeted therapies; furthermore, as in other cancers, ROS/RNS and antioxidant systems show two-faced roles, often in relation to the different stages of disease progression [76,77,78,79].

In the next two paragraphs, we discuss the main and growing body of recent evidence for oxidative stress-related pathways in cancer disease, focusing on melanoma and its acquired resistance to targeted therapies.

## 3. Oxidative Stress in Melanoma

### 3.1. ROS/RNS in Melanoma

#### 3.1.1. Reasons for ROS/RNS Increase

A growing body of evidence indicates that compared with normal healthy tissue, tumor tissues, including melanoma, exhibit a high level of ROS/RNS [68,80,81]. Several reasons can explain this feature through both environmental and internal mechanisms. Cigarette smoke and ultraviolet (UV) radiation are certainly among the primary external causes of ROS increasing [82,83]. In terms of internal mechanisms, ROS production is enhanced in cancer cells as a result of the activation of several well-known oncogenes, loss of tumor suppressors [84,85], tumor hypoxia [84,86], altered integrin signaling [87], and reprogrammed metabolism [84,88].

**Figure 1:**
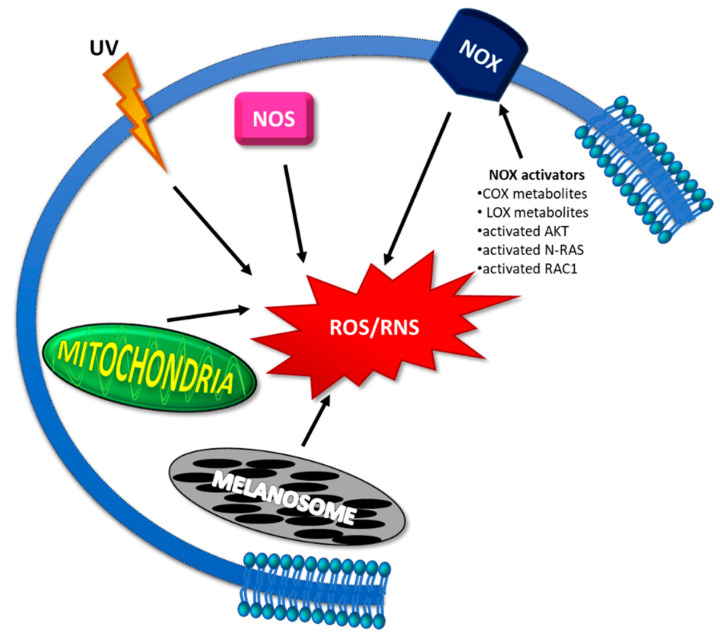
Major ROS sources in melanocytes. ROS can be generated from UV irradiation, melanosomes, mitochondria, and NOS and NOX family enzymes. Moreover, NOX enzymes can be activated by arachidonic acid metabolites from both the COX and LOX pathways, as well as from the activated N-RAS, AKT, and RAC1 oncogenic pathways.

In comparison with other solid tumors, ROS levels are particularly elevated in melanomas [89] (Figure 1). Two important tissue characteristics may explain this further increase: the natural exposure to UV radiation and the presence of melanin [90].

UV irradiation, a major contributor to skin cancer, can directly damage DNA via forming a large amount of cyclobutane pyrimidine dimers (CPDs), pyrimidine (6-4) pyrimidone adducts [79]; moreover, UV induces the skin to produce, through photosensitizer molecules, high levels of ROS (singlet oxygen and hydroxyl radicals) immediately after irradiation and of RNS (NO and possibly ONOO^−^) at later timepoints [91,92]. Free radical enhancements can also originate from UV-dependent activation of ROS-producing enzymes, such as NOXs [93].

Another important source of ROS in the skin is melanin synthesis, which involves oxidative reactions, with the production of superoxide anion and hydrogen peroxide (H_2_O_2_) at various steps of the synthetic melanin pathway [88]. Moreover, melanin has a two-faced role in determining oxidative stress levels: on the one hand, it absorbs UV radiation, thus protecting skin cells from oxidative damage; on the other hand, it shows pro-oxidant activities [94]. This double role can be partially explained by considering the different pro-oxidant activity of the two main forms of melanin present in the skin: the brown-black eumelanin and reddish-yellow red pheomelanin. Indeed, red pheomelanin-dependent ROS formation has been reported to occur with or without UV radiation [95], while black eumelanin, if present in sufficient amounts, can counterbalance this production. Thus, higher levels of pheomelanin can indirectly contribute to mutagenesis through enhanced ROS production [96]. Furthermore, the human melanocortin 1 receptor gene (MC1R) wild type signaling, which is responsible for black eumelanin production [97], also promoted DNA repair and ROS scavenging [98]; loss-of-function variants of MC1R associated to pheomelanin production [97] elicited enhanced ROS production and impairment of DNA repair [98], likely contributing to the observed higher rate of melanoma onset.

In cancer cells, the major source of ROS/RNS has been attributed to the dysfunction of mitochondrial respiratory chain enzymes [99]; however, in melanoma, other enzymes seem to play a major role in ROS production: the NOX family, nitric oxide synthases (NOSs), arachidonic acid cyclooxygenases (COXs), and lipoxygenases (LOXs) [76,94].

The ROS-producing NOX enzymes are complex multidomain proteins with different distribution in tissues and cellular sublocalization (plasma membrane, cytoplasm, nuclear membrane, mitochondrial, ER membranes). The first identified member was NOX2, present in the phagocytic vacuole of “professional” phagocytes; later, six additional members were found (NOX1, NOX3, NOX4, NOX5, DUOX1, DUOX2) [100]. Among them, NOX1, NOX4, and NOX5 have been found to be expressed in the melanocytic lineage [76]. Interestingly, early studies suggested that NOXs contribute to melanoma cell proliferation [101]. Later, emerging evidence indicated that specific NOX isoforms may impact both melanomagenesis and melanoma progression [76]. NOX1 was found to be overexpressed in melanoma cell lines, and its ability to enhance cell invasion by matrix metalloproteinase-2 upregulation and EMT induction was demonstrated [102]. NOX1 can be activated by the small GTPase Ras-related C3 botulinum toxin substrate 1 (RAC1) [103], a Rho GTPase family protein involved in the regulation of migration with a recognized role in cancer metastasis [104]. Mutated N-RAS also can stimulate NOX1 to produce ROS in melanocytes [77]. Moreover, the specific NOX1 inhibitor GKT771 decreased B16F10 melanoma cell proliferation *in vitro* and suppressed B16F10 tumor growth and blocked angio/lymphangiogenesis *in vivo* [105]. However, despite its well-demonstrated proinvasive role in preclinical studies, NOX1 expression is not associated with melanoma progression, since there were reportedly similar levels in early-stage noninvasive primary tumors and MM human samples [76]. On the contrary, NOX4 was found to be highly expressed in metastatic samples as compared to in early-stage primary tumors [76]. NOX4 contributes to the transformed phenotype of melanoma cells by regulating G2–M cell cycle progression [106]. It is also a downstream target of AKT, a serine-threonine kinase frequently activated in melanoma [107]; in particular, it has been proposed that the ROS generated by the AKT-activated NOX4 contribute to the transformation of radial growth to vertical growth required for the acquisition of the invasive and metastatic phenotype [108]. Interestingly, it has been demonstrated that ROS produced by NOX4 are able to coordinate cell survival through the focal adhesion kinase (FAK) pathway, thus maintaining cell adhesion and viability [109]. NOX5 is upregulated in melanoma as well as in other cancer types [110]. Moreover, it has been demonstrated that NOX5 can affect cell proliferation partially through ROS extracellular production and is able to modulate several signaling pathways, such as HIF-1α and p27Kip1 [110].

In melanomas, besides NOX, other enzymes can also contribute significantly to oxidative stress increase, such as the nitric oxide synthase (NOS) family, which can synthesize RNS such as NO [76,111]. Superoxide produced by NOX may react with NO, which is produced by NOS, thereby generating the peroxynitrite ONOO^−^. All the three isoforms, neuronal (nNOS), inducible (iNOS), and endothelial (eNOS), have been demonstrated to be involved in melanoma progression. iNOS and nNOS promote melanoma cell proliferation [112,113]; nNOS has a role in melanoma invasion [112]; iNOS is associated with increased resistance to cisplatin [114] and poor patient survival [115]; and eNOS is involved in lymphangiogenesis and lymphatic metastasis [116]. In some cases, eNOS catalyzes the reduction of O_2_ to O_2_^•−^ instead, a phenomenon that is usually described as uncoupling [117]. In addition, peroxynitrite, in turn, has been shown to uncouple eNOS, thereby converting eNOS to a superoxide-producing enzyme. As a consequence, NO production is reduced, and the oxidative stress, in particular represented by both the O_2_^•−^ and ONOO^−^ species, further increases [118,119,120]. Interestingly, it has been demonstrated that eNOS uncoupling, by eliciting superoxide overproduction, can drive malignant melanoma transformation [119]. Moreover, a high level of nitrotyrosine, an indirect biomarker of NO, was found to be significantly associated with poor survival of melanoma patients [115].

It has been demonstrated that arachidonic acid metabolites from both COX and LOX pathways may activate ROS production via NOX stimulation [120]. Interestingly, inhibitors of both COX and LOX pathways convert mouse melanoma to a noninvasive phenotype by downregulating matrix metalloproteinase-2 (MMP-2) [121], an enzyme able to degrade the basement membrane components that can be activated by intracellular ROS/RNS [122]; thus, COX and LOX inhibitors could possibly reduce invasiveness by lowering ROS levels, which in turn would downregulate MMP-2 activity.

#### 3.1.2. Consequences of ROS/RNS Increase

Enhanced ROS production triggers the occurrence and development of melanoma through genotoxic and nongenotoxic pathways.

At the nuclear level, ROS elicit DNA oxidative damage by generating 7,8-dihydro-8-oxo-2′-deoxyguanosine (8-oxodG) and by producing DNA double-strand breaks (DSBs), leading to genomic instability [123]. 8-oxodG is considered a premutagenic DNA lesion, since during DNA replication, it can cause a dC:dG to dA:dT transversion [124]. In agreement with its mutagenic role, 8-oxodG was demonstrated to be lower in melanoma patients with significantly longer survival time than in those with shorter survival time [125]. Moreover, the genotoxic pathway includes epigenetic modifications, in particular eliciting a global hypomethylation of the genome and aberrant CpG island hypermethylation of some genes [79]. Thus, through direct mutagenesis or epigenetic dysregulation of gene expression, protooncogenes such as BRAF, N-Ras, and RAC1 can be activated, while tumor suppressors like p53, protein patched homolog 1 (PTCH1), and phosphatase and tensin homolog (PTEN) can be inactivated [79].

In addition, ROS have a nongenotoxic protumoral effect by modulating a number of oxidative-stress signaling pathways, as well as antioxidant pathways, such as MAPK, PI3K/protein kinase B (PKB), AKT/mammalian target of rapamycin (mTOR), Nrf2, and nuclear factor-κB (NF-κB) [77,79,126].

Interestingly, ROS produced by melanoma cancer cells or exogenously by UV irradiation also have the ability to modulate functions of noncancer cells surrounding the tumor, such as cancer-associated fibroblasts (CAFs) and tumor-infiltrating T-cells (TITL) [127]. Moreover, other cells of the tumor microenvironment (TME), such as tumor-associated macrophages (TAMs) or inflammatory cells, also produce ROS, which in turn can affect the functioning of immune and cancer cells [128]. This complex interplay between ROS produced by tumor cells and by cells of the TME regulates melanoma progression, drug resistance, and immunosurveillance [127].

#### 3.1.3. Enhancing ROS Production as Melanoma Anticancer Therapy

Several chemotherapeutics trigger cancer cell death by increasing ROS production. High, lethal levels of ROS can be generated by anthracyclines (e.g., doxorubicin, daunorubicin, and epirubicin), platinum-based complexes (e.g., carboplatin, cisplatin, and oxaliplatin), camptothecins (e.g., irinotecan and topotecan), epipodophyllotoxins (e.g., etoposide), and alkilant agents (dacarbazine, temozolomide-TMZ, carmustine, fotemustine), while taxanes (e.g., paclitaxel and docetaxel) and vinca alkaloids (e.g., vincristine and vinblastine) generate low ROS levels [129]. These conventional pro-oxidant drugs are scarcely effective in melanoma; however, some classes of ROS inducers have attracted the attention of scientists. This is the case for photosensitizing chemical substances (Ps). They belong mainly to the porphyrin, phthalocyanine, chlorin, and porphycene classes, and most of them are of plant origin. Ps have been successfully employed in photodynamic therapy (PDT), which has been investigated for the past 30 years as an unconventional and alternative treatment for cancer [130]. Ps are nontoxic dyes, but when excited by visible light, they can produce ROS at lethal doses able to kill cancer cells [130]. Although melanoma can benefit from this approach, with the employment of Ps such as verteporfin, 5-aminolevulinic acid (5-ALA), and others in preclinical studies [131], its clinical use to treat *in situ* melanoma is still controversial.

Another extremely attractive strategy consists in the use of pro-oxidant drugs loaded in nanoparticles. Indeed, the employment of nanotechnology is extremely attractive in cancer drug delivery mechanisms. Drug-loaded nanoparticles offer several advantages: prolonging drug half-life, enhancing drug localization, increasing drug efficacy, minimizing toxic side effects, and diminishing the chances of multidrug resistance [132]. Melanoma also can benefit from these advantages [133,134,135]. Thus, treatments with old pro-oxidant drugs, such as paclitaxel, dacarbazine, doxorubicin, but also Ps loaded in nanoparticles, being more effective, become more appealing for clinical treatments [133,134,135,136]. Recently, the nanoparticle-albumin-bound paclitaxel (Nab-Paclitaxel), indicated as monotherapy or in combination with other drugs for the treatment of advanced breast, pancreatic, and non-small cell lung cancers [137], has also gained attention for MM treatment [138]. In particular, a recent clinical trial showed that ipilimumab immunotherapy followed by chemotherapy with Nab-Paclitaxel and the antiangiogenic bevacizumab for the treatment of BRAF wild-type MM revealed a favorable effect [139].

Finally, novel natural ROS-inducer compounds derived from plants or animals have been continuously under study in preclinical research. For example, cantharidin, a terpenoid isolated from the insect mylabris (*Mylabris phalerata Pallas*), and Withaferin A (WFA), a withanolide derived from the medicinal plant *Withania somnifera*, have demonstrated antitumorigenic activity in melanoma through the generation of ROS [140,141].

### 3.2. Antioxidant Systems in Melanoma

To maintain acceptable sublethal ROS levels, which allow tumorigenic phenotype maintenance, cancer cells, including melanoma, usually increase their antioxidant systems to protect cells from oxidative stress damage and favor their survival. However, this adaptative process seems to elicit the rise of more resistant cell subclones [59,60].

Below, the main antioxidant defenses in melanoma under the control of the transcription factor Nrf2, together with other enzymatic and nonenzymatic pathways, are reported.

#### 3.2.1. Nfr2 and Its Signaling Pathway

Initially, a protective role for Nrf2 was recognized in the early stages of malignant transformation because of its ability to detoxify carcinogens and protect cells from oxidative stress damage. However, Nrf2 in the advanced stages of the cancer disease has an opposite role, since its activation has been shown to be involved in modulating cell proliferation, EMT, migration, invasion, angiogenesis, and tumor progression [63,142] as well as chemoresistance and radioresistance of various malignant tumors [143,144].

Nrf2 has also been found to have a critical role in disease progression in melanoma. In a cohort of 36 nevi, 14 lentigo maligna, and 71 malignant melanomas, Hintsala and collaborators [145] demonstrated that nuclear Nrf2 expression correlated with deeper Breslow depth, invasive phenotype (Clark III–V), nodular growth, and worse melanoma-specific survival. These authors suggested that Nrf2 could offer melanoma cells a growth advantage, allowing them to better survive in a hostile oxidative stress condition. These results were later confirmed in larger cohorts of patients, where it was demonstrated that Nrf2 expression was higher and cytoplasmic Keap1 lower in metastatic lesions than at primary sites [146] and that nuclear Nrf2 expression was correlated with a significantly worse survival rate [147].

Moreover, the inhibition of Nrf2 with siRNA or other genetic or pharmacological strategies elicits a wide range of antitumoral responses in experimental models of melanoma both *in vitro* and *in vivo*. Nrf2 downregulation inhibited melanoma cell proliferation, migration, and invasion [148,149], induced apoptosis [148], enhanced sensitivity toward oxidative stress [149] and ionizing radiation [148], and reverted chemoresistance to cisplatin, dacarbazine [150], and TMZ [151]. Apparently in contrast with this evidence, Zhu and collaborators [152] demonstrated that Nrf2 deficiency promoted melanoma growth and lung metastasis when B16-F10 cells were inoculated in Nrf2-null C57BL/6 mice. With the aim to explain their findings, these authors suggested that a possible mechanism could be related to the dysregulated immunity observed in Nrf2-null mice, which can have a profound impact on the progression of the disease.

The mechanisms leading to Nrf2 activation in melanoma need to be fully elucidated. Somatic Nrf2 gain-of-function and Keap1 loss-of-function mutations are frequent in tumors and correlate with chemo/radioresistance and poor clinical outcome [153]; however, even if Keap1 missense or nonsense mutations have also been reported [150], in melanoma, Keap1 and Nrf2 mutations are not frequent.

Recently, in a large cohort of melanoma tumor samples with different degrees of malignancy, it was demonstrated that there was no correlation between immunostaining of the Nrf2 protein and mRNA expression levels [147]. Thus, very likely, post-translational mechanisms can account for the Nrf2 activation in melanoma [73]. Among these mechanisms, short noncoding RNAs, in particular microRNAs (miRNAs), are gaining attention for their ability to control gene expression. Their levels are dysregulated in melanoma, which contributes to disease development and resistance to target therapy [154]. Recent evidence has shown a negative correlation between miRNA controlling Nrf2 mRNA levels (miR-23B, miR-93, miR-144, and miR-212) and the protein expression of Nrf2 [147]. Moreover, recent findings demonstrated that BRAF-mutated cells could upregulate Nrf2 protein through the activation of FAM129B, an antioxidant protein also known as Niban-like protein 1 or MINERVA. In particular, in BRAF V600E mutant melanoma, the MAPK activated pathway leads to hyperphosphorylation of FAM129B, which in turn can compete with Nrf2 for the binding to Keap1; in the absence of its inhibitor Keap1, Nrf2 protein is more stable; thus, its level can increase [155].

Several Nrf2 downstream genes can also contribute to chemoresistance and tumor progression.

HO-1 is an enzyme able to degrade the pro-oxidant free heme into carbon monoxide, ferrous iron, and biliverdin which is quickly transformed into bilirubin. The end-products of HO-1 have antioxidant activities, and HO-1 overexpression has been found in various tumor types, facilitating tumor growth, cancer progression, and drug resistance [156]. In melanoma, overexpression of HO-1 resulted in enhanced cell proliferation, resistance to hydrogen peroxide-induced oxidative stress, and endothelial cell division contributing to angiogenesis [157]. These results have also been confirmed *in vivo* by injecting HO-1 overexpressing melanoma cells in mice, compared to mice injected with wild-type melanoma cells [157].

Peroxiredoxins (PRDXs) are non-seleno peroxidases that catalyze the reduction of a broad spectrum of peroxides. There are six of these enzymes (PRDX 1–6), and they present thiol groups of their cysteine (Cys) as catalytic centers. PRDX 1–5 contain two conserved catalytic Cys and utilize thioredoxin as a reductant [158]. Conversely, PRDX6 contains a single conserved Cys and utilizes glutathione (GSH) but not thioredoxin to catalyze the reduction of H_2_O_2_ and other organic peroxides [158]. PRDX6 seems to be the only PRDX able to also reduce phospholipid hydroperoxides (PLOOH) [159]; moreover, PRDX6 is a bifunctional enzyme, having both peroxidase and phospholipase A2 activities. The latter is involved in physiological lung function, by contributing to the production of surfactant, as well as in lung cancer metastasis [160]. Both PRDX1 and PRDX6 are downstream targets of Nrf2 [161], and, as with other peroxiredoxin isoforms, they are frequently elevated in several human cancers [162,163,164,165,166]. A high level of PRDX6 can be found in most melanoma cells. Its expression levels are post-transcriptional maintained, particularly by EGFR signaling. Moreover, it has been demonstrated that PRDX6 enhances cell viability mainly by enhancing proliferation and that this action is mediated by its phospholipase A2 activity. In particular, PRDX6 exerts its effect by enhancing the production of arachidonic acid (AA) [167]. PRDX6 can also be involved in tumor progression, since it was shown that its expression correlated with melanoma malignancy [168]. Interestingly, other PRDX not controlled by Nrf2, such as PRDX2, can be correlated with melanoma malignancy [168].

GSTs are a family of detoxification enzymes able to conjugate reduced GSH to a wide number of exogenous and endogenous electrophilic and hydrophobic substrates, participating in tumorigenic processes and drug resistance [169]. Among them, GST alpha (GSTA1-A4), mu (GSTM1-M4), and pi (GSTP1) are the downstream targets of Nrf2 [161,170,171]. Notably, GSTA4 is able to detoxify the lipid peroxidation product HNE [172]. In melanoma, it was shown that GSTM3 expression correlated with melanoma malignancy [168]. Moreover, the GSTP1 polymorphism rs1695, which encodes the amino acid change p.Ile105Val, was associated with metastatic disease [173].

Other Nrf2 downstream targets have been found involved in melanoma progression. TRX was found to be overexpressed in human melanoma and is positively associated with metastasis [174]; studies using human tumor biopsy samples demonstrated that overexpression of xCT was correlated with melanoma stage and progression, and xenograft studies confirmed the ability of xCT overexpression in melanoma cells to lead to more aggressive tumors compared to vector controls [175], an opposite role from that of a member of the GPX family, GPX3, which has been found to correlate to poor prognosis in melanoma [176].

#### 3.2.2. Other Antioxidant Enzymes and Molecules

One of the main enzymatic components of the antioxidant defense system is represented by the metal ion-dependent superoxide dismutases (SODs), which can convert superoxides to hydrogen peroxide, which is further removed by catalase (CAT) and glutathione peroxidase (GPx). There are three members of the SOD family, with different subcellular localization: (i) the cytosolic copper/zinc SOD (CnZnSOD or SOD1), which is present in the nucleus and mitochondrial inner membrane; (ii) the mitochondrial manganese-dependent SOD (MnSOD or SOD2); and (iii) the extracellular SOD, which also contains Cn/Zn (EcSOD or SOD3). All three enzymes are involved in the development of cancer, but with different roles, which are often controversial [177,178,179]. Little is known about cytosolic CnZnSOD in melanoma, while extensive studies on MnSOD have demonstrated that it is involved in melanoma cancer growth control and tumor progression. Overexpression of MnSOD promotes the survival of melanoma cells exposed to the cytostatic and cytotoxic effects of cytokines (interleukin-1, IL-1; tumor necrosis factor, TNF), chemotherapeutic agents (doxorubicin, mitomycin C), and ionizing irradiation [180]. Moreover, overexpression of MnSOD may promote the survival of melanoma cells exposed to the chemotherapeutic drug doxorubicin [181]. Interestingly, MnSOD activity has been found to parallel disease progression, since it progressively rises to start from the early stage until the metastatic phenotype [182]. In contrast with these findings, Church and collaborators demonstrated that increased SOD2 expression suppressed the malignant phenotype of human melanoma cells [183].

An opposite role was found for EcSOD. Indeed, in melanoma and in other tumors, it has an antiproliferative role, suggesting that the loss of extracellular redox regulation creates a microenvironment favorable to cancer progression [179].

Conflicting results have been obtained about the role of catalase in melanoma. On the one hand, it was shown that catalase overexpression correlated with regression of melanoma malignancy and that its downregulation could favor malignant progression [184]. On the other hand, it was demonstrated that catalase activity increased with disease progression, achieving the maximum in stage III [182].

#### 3.2.3. Targeting Redox Homeostasis as Melanoma Anticancer Therapy

Given the role of ROS in cancer initiation and tumor progression, the use of antioxidants as therapeutical agents is extremely appealing in melanoma.

Dietary polyphenols, a broad spectrum of plant-derived compounds with antioxidant properties, have shown antimelanoma effects in several *in vitro* and *in vivo* experimental models [185]. For instance, fisetin could reduce melanoma tumor growth in mouse xenografts [186]; honokiol, a NOX1 inhibitor, by reducing cellular ROS levels, decreased the migratory potential of melanoma cells in an *in vitro* model [187]; and anthocyanins could inhibit proliferation, increase oxidative stress, and reduce mitochondrial membrane potential in melanoma cells but not in normal cells [188]. However, the clinical applications of polyphenols as chemopreventive or therapeutical agents are strongly limited by low bioavailability, since these plant-derived nutrients undergo important degradative or catabolic processes due to the gastrointestinal transit, including metabolic processation from the intestinal microbiota [185]. To overcome these limitations, the use of polyphenol-loaded nanocarriers seems to have effective clinical potential in melanoma [189].

Other natural, such as the pentacyclic triterpenoid lupeol [190] or synthetic, such as the specific iNOS L-N6-(1-Iminoethyl)lysine (L-NIL) [114], antioxidants also have been studied with the goal to fight melanoma.

However, it is highly simplistic to consider ROS as promoters of cancer initiation and progression and antioxidants as anticancer agents. In some cases, antioxidants used to fight melanoma in *in vivo* animal studies or in human clinical trials not only failed to stop the disease but even showed the ability to favor it, proving to be a double edge sword.

This two-faced characteristic has been shown, for instance, for N-acetylcysteine (NAC). This antioxidant has been shown to protect melanocytes against oxidative stress/damage and delay onset of UV-induced melanoma in mice [191]; furthermore, it can be safely administered to patients at increased risk for melanoma, since 3 h after ingestion, NAC attenuated GSH-depletion was induced by UV in nevi, suggesting its possible use prophylactically before acute UV exposure with the ultimate goal to reduce long-term melanoma risk [192]. However, in an endogenous mouse model of malignant melanoma, NAC did not impact melanoma cell proliferation but also increased lymph node metastases and GSH intratumoral levels [193]. In support of the hypothesis that antioxidants may not have efficacy or even be detrimental *in vivo*, several clinical trials failed to show anticancer activity. For example, a meta-analysis of nine randomized controlled trials and prospective observational studies concluded that the consumption of A, C, and E vitamins, selenium, and carotenoids as food (fruits and vegetables), supplements, or both did not reduce the incidence of cutaneous melanoma [194]. Moreover, Piskounova and collaborators studied the oxidative stress status of several melanoma circulating cells and their ability to metastasize when xenografted into NOD/SCID IL2Rγnull (NSG) mice [195]. Interestingly, they found that oxidative stress on circulating melanoma cells inhibited distant metastases, while antioxidants promoted it.

**Figure 2:**
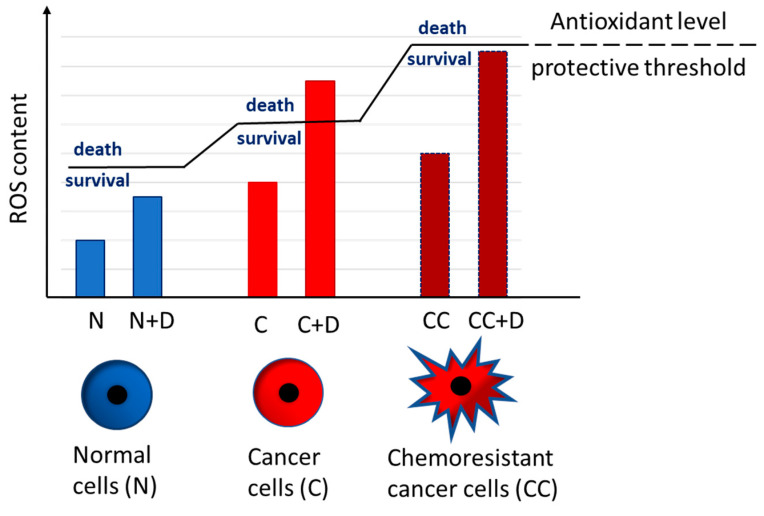
ROS levels and antioxidant threshold. Normal cells (N), cancer cells (C), and chemoresistant cancer cells (CC) can have different susceptibility to therapeutical approaches with drugs able to induce ROS production (drug, D). Low levels of ROS are required for cell survival, and medium levels are tolerated; however, overwhelming levels of ROS trigger cell death. N have low ROS, fully balanced by a robust antioxidant system. When ROS are increased by drug treatment, normal cells generally survive, being protected by antioxidants. Conversely, C have increased levels of basal ROS compared to normal cells. Moreover, C undergo a redox adaptation enhancing their antioxidant defenses; nevertheless, when ROS are increased by therapeutic approaches, they reach the death threshold earlier, and tumor cells can be killed more easily. In CC, ROS can be higher than in C; moreover, a consistent redox adaptation leading to an increased expression of antioxidants can be observed. In these conditions, ROS-inducing drug treatment may fail to kill cancer cells. The use of a combination of drugs that simultaneously induce new ROS generation and inhibit antioxidant defenses seems to be most promising.

These observations have led to a radical change of perspective, supporting treatments that inhibit antioxidants and thus favor the accumulation of lethal ROS levels. Indeed, by blocking antioxidant defense in tumors, it is possible to decrease their ability to balance oxidative insult, eliciting cell death (Figure 2).

Several synthetic and natural compounds can sensitize cancer cells to oxidative stress induced by chemo- and radiotherapy by blocking their antioxidant defense [196]. L-buthionine [S,R]-sulfoximine (BSO), a synthetic inhibitor of the γ-glutamylcysteine synthetase, a key enzyme in glutathione biosynthesis, can revert chemoresistance to TMZ both *in vitro* and *in vivo* [151]. Among natural compounds, Nrf2 inhibitors have gained the attention of scientists [64]. Cotreatment with the quassinoid brusatol, a plant-derived Nrf2 inhibitor, and low-dose UVA irradiation increased intracellular ROS, inhibited melanoma cell proliferation, and induced cell apoptosis *in vitro* and *in vivo* [197]. Ailanthone, a plant extract derived from the tree *Ailanthus altissima* that can downregulate Nrf2 and induce oxidative stress [198,199,200], has shown anticancer activity toward a wide range of chemoresistant tumor cells [199,200,201,202,203], including melanoma [204]. Resveratrol, a natural polyphenol, can decrease Nrf2 expression in melanoma cells, eliciting ROS increase and inhibition of growth and proliferation by downregulating the Bcl-2 protein level and upregulating Bcl-2-related X protein expression [205]. The flavonoid Nrf2 inhibitor Luteolin, present in various vegetables and fruits, efficiently inhibited GST, leading to GSH depletion in melanoma cells [206]. MC3165 and MC3181, two nitrobenzoxadiazole (NBD) analogs that are orally active and water-soluble, specifically target GSTP1 and can inhibit viability in a panel of human melanoma cell lines and in several human melanoma xenograft models [207].

### 3.3. Lipid Peroxidation in Melanoma

LPO can be described generally as a process under which oxidants, ROS/RNS, or nonradical species attack lipids containing carbon–carbon double bond(s), especially of PUFAs, leading to the formation of lipid radicals (L^•^). Following this first phase of initiation, the L^•^ rapidly react with oxygen to form lipid peroxy radicals (LOO^•^), which can further propagate oxidative degradation by forming new L^•^ (which continue the chain reaction) and lipid hydroperoxides (LOOH) (propagation step). In the last step, the termination phase, antioxidants such as vitamin E donate a hydrogen atom to lipid peroxy radicals, forming nonradical products [44,208]. The highly unstable LOOH can further generate new peroxyl and alkoxy radicals and decompose to secondary products, which have been considered “oxidative stress second messengers” because of their ability to diffuse from their site of formation and prolonged half-life compared to that of free radicals. These secondary lipid peroxidation products are mainly composed of reactive aldehydes, such as MDA, HNE, hexanal, and acrolein, and have been extensively studied for their surprising and sometimes unexpected biological activities [45,51,61,66,209]. HNE, one of the most active lipid peroxidation products, can easily react with both low-molecular-weight compounds and macromolecules, such as proteins and DNA. HNE–DNA adducts could contribute to the mutagenic and carcinogenic effects associated with oxidative stress-induced LPO; not only are HNE–protein adducts markers of LPO, but the covalent modifications of proteins can profoundly modify their biological activity, contributing to the complex biological consequences observed in tissues exposed to ROS [53,55]. MDA, one of the most used markers of LPO in tissues, and acrolein also have high capabilities to react with many biomolecules such as proteins or DNA, leading to the formation of adducts [61,210].

Although an increase in oxidative stress has been demonstrated in the majority of tumor types, the lipid peroxidation product content in cancer cells has been found not only to increase but to decrease also. Since the results have been contradictory, the role of LPO and its products in cancer development is a matter of debate.

First investigations in this field demonstrated that the levels of LPO products in hepatoma cells were lower than those found in normal liver cells; that they strongly decreased in mostly highly dedifferentiated hepatoma; and that, in the model of diethylnitrosamine carcinogenesis, the LPO decline occurred as early as at the stage of reversible nodules and progressed until the development of clear hepatomas [211,212,213]. Accordingly, HNE–protein adducts were found in lower levels, with respect to their correspondent physiological conditions, in the kidney [213] and in colon cancer, in which this decline paralleled the histological degree of dedifferentiation [214]. On the contrary, other experimental results demonstrated that HNE and MDA contents were higher than in normal tissues in colorectal cancer [215], thyroid tumors [216], invasive breast carcinomas [217], and astrocytic and ependymal cancer, with increasing levels at higher grades of malignancy [218]. Probably, this discrepancy can be ascribed to different levels of PUFAs, in particular arachidonic and linoleic acids, the major substrates for lipid peroxidation, in the tumor cell membranes.

In melanoma, the data seem to agree with these latter findings. Indeed, MDA levels were significantly higher in human melanoma tissues than in control tissues [219]. Several studies also confirmed increased MDA concentration in the plasma of melanoma-bearing mice [220] and in human melanoma patients [182]. Interestingly, not only are serum MDA levels elevated in all stages of melanoma, but stage IV patients showed the highest contents compared to other stages [182]. In a cohort of 5 simple nevi, 5 dysplastic nevi, 35 primary malignant melanomas, and 10 metastases, Blendea and collaborators [221] performed HNE immunostaining of patients’ tissues. Interestingly, they found that HNE content was significantly increased in dysplastic nevi versus benign nevi. It was maintained at a level comparable to that in dysplastic lesions in cutaneous malignant melanomas, while it was lost in metastases. The authors suggested that HNE is involved early in the process of melanoma tumorigenesis and that the HNE loss in metastases could correlate with the increased proliferative activity of metastatic cells. Indeed, at micromolar concentrations similar to those found in human plasma and tissues, HNE has shown antiproliferative, proapoptotic, antiangiogenic, and prodifferentiative actions in a wide variety of tumor cells *in vitro* by modulating the expression of critical genes for cancer, such as oncogenes, oncosuppressors, transcription factors, apoptotic genes, and miRNAs [53,56,66,222,223,224]. Prof. Dianzani and Prof. Esterbauer were pioneering researchers on HNE signaling. In particular, they were the first to demonstrate that HNE could negatively regulate cell tumor growth by affecting the expression of oncogenes such as c-myc, c-myb, and c-fos [225,226,227], either acting alone or interacting with growth factors present in serum [228].

This anticancer activity has also been widely demonstrated in melanoma cells. Early studies demonstrated that HNE treatment inhibited B16-F10 melanoma cells both *in vitro* and *in vivo* [229]. Moreover, it has been demonstrated that the treatment of pigmented murine melanoma B16-F10 cells and amelanotic murine melanoma B16BL6 cells with toxic doses of HNE rendered surviving cells more resistant to oxidative stress, possibly by forming a bioactive conjugate with an extracellular peptide/protein present in the serum media. This conjugate was supposed to be responsible for the suppression of cancer growth exerted by HNE and was observed only in the presence of serum [230]. Later, in an attempt to exploit the antitumoral HNE properties *in vivo*, strategies based on the use of nanovehicles able to favor the delivery of this extremely reactive and poorly soluble aldehyde have been proposed. Both β-cyclodextrin-poly(4-acryloylmorpholine) conjugate and β-cyclodextrin-based lipid nanocapsules loaded with HNE were shown to potentiate its antitumor effects, including in melanoma cells. Interestingly, successful topical administration of these HNE-loaded nanovehicles on a three-dimensional human reconstructed model of skin melanoma encouraged a possible clinical application [231,232].

On the basis of these results, which have demonstrated the role of LPO products in the control of cancer growth, some strategies have been developed to target enzymes involved in LPO product detoxification.

### 3.4. Enzymatic Systems Detoxifying LPO Products in Melanoma

Aldehyde dehydrogenases (ALDHs) are a superfamily of NAD+- or NADP+-dependent enzymes responsible for most of the metabolism of aliphatic and aromatic, exogenous, and endogenous aldehydes. In humans, ALDHs have been divided into 19 different families that, in turn, include various numbers of subfamilies. Members of families show different tissue and subcellular localization and specific functions. In light of the differences in substrates metabolized, ALDHs can play the role of detoxifying enzymes towards toxic aldehydes, contribute to the synthesis of molecules requiring the metabolism of an aldehydic intermediate, or modulate crucial cellular functions such as proliferation, differentiation, and survival. Interestingly, all ALDH isoenzymes, and in particular cytoplasmatic ALDH1 and ALDH3, are involved in the catabolism of aldehyde derived from lipid peroxidation of PUFAs [233].

Based on the above-cited activities, changes in ALDH expression in several types of tumors have been deeply investigated in experimental models of carcinogenesis, cancer cell lines, and neoplastic patients. A direct correlation between increased ALDH expression and malignancy degree has been reported in diethyl-nitrosamine-induced hepatocarcinogenesis in rats and in rat hepatoma cell lines [234,235]. In human cancers, ALDH1A1 and ALDH1A3 have been found to be increased and correlated with some parameters of cancer staging, the ability of cancer cells to form metastases, and the onset of resistance to chemotherapeutic drugs [236,237,238,239].

With regard to the latter aspect, the enhancement of ALDH expression is important mainly in favoring the resistance of cancer cells to drugs acting through the production of ROS, such as oxazaphosphorines, doxorubicin, and TMZ, the metabolic intermediates of which are catabolized by ALDHs [240,241,242].

More recently, ALDHs have also been included in the markers of cancer staminal cells (CSCs) and ALDH+ subpopulations, showing that other CSC characteristics, including self-renewal and differentiation, have been isolated from different types of tumors, including melanoma [243,244,245,246].

High expression of ALDHs was observed in xenografted melanoma from human cancers and in xenografted human MM, even if, in the latter study, no differences in tumorigenicity and drug resistance between ALDH+ and ALDH- subpopulations were reported [247,248]. The possibility that ALDH+ cells can be considered as a marker of melanoma initiating cells (MICs) has been suggested.

Among ALDH isoenzymes, ALDH1A1 and ALDH1A3 were more associated with CSC phenotype, even if the results present in the literature have been contradictory. Lou and colleagues [235] reported that both members of the ALDH1 family are increased in human melanoma cell lines, whereas more recently, Pérez-Alea and colleagues [249] indicated ALDH1A3 as the enzyme majorly expressed in cultured melanoma cells. The same authors also evidenced that ALDH1A inhibition and ALDH1A3 depletion both caused apoptosis induction in melanoma cells.

Though full understanding of ALDH expression in melanoma and in melanoma cells would require yet further investigations, the importance of these enzymes, mainly in modulating cell resistance to anticancer drugs, has aroused great scientific interest. With the aim of exploiting these properties, several direct or indirect approaches have been used, including silencing and specific inhibitors. siRNA against ALDH1A1 or ALDH1A3 has been shown to revert resistance to TMZ and paclitaxel [247]. In a similar way, the direct blocking of ALDH by the specific inhibitors dacarbazine, disulfiram, and diethylaminobenzaldehyde reduced the number of residual tumorigenic melanoma cells after chemotherapeutic treatment [250]. It has been suggested that the beneficial effect of ALDH1 inhibition on drug resistance could be due to the negative modulation of the Hedgehog signaling pathway involved in maintaining MIC [251], of drug pump ABCB1, and of proteins showing antiapoptotic effects [252].

Other than canonical ALDH inhibitors, new potential molecules have been synthesized. Dinavahi and colleagues prepared multi-ALDH inhibitors showing an isatin backbone able to cause toxic intracellular aldehyde accumulation, cell cycle arrest, and apoptosis [253].

Notably, in the case of some types of anticancer drugs, high ALDH expression can represent a favorable condition. For example, 5-nitrofuran, an antibiotic prodrug showing anticancer potential, is activated to cytotoxic metabolite by ALDH1 isoforms, thus rendering melanoma cells highly expressing ALDHs more susceptible to eradication [254].

Other than as a marker of CSC, ALDH1A1 and ALDH1A3 expression has been indicated as a marker of responsivity to BRAF/MEK inhibitors in BRAF-mutant MM patients [255]. This recent finding further amplifies the clinical implications of ALDH evaluation and modulation in patients bearing melanoma.

## 4. Oxidative Stress in Resistance to Targeted Therapies in Melanoma

The presence of the BRAFV600E mutation has been associated with regulating redox homeostasis by enhancing glycolytic metabolism and lowering mitochondrial oxidative phosphorylation (OXPHOS); however, after the initial cancer regression with BRAFi/MEKi therapies, a subclone of melanoma cells can acquire a drug-resistance phenotype characterized by the enhancement of mitochondrial biogenesis, activity, and content, leading to a further increase in mitochondrial ROS production [256,257]. This metabolic rewiring, from glycolysis toward OXPHOS, can be considered an adaptative response that allows melanoma cells to produce sufficient ATP levels to survive despite the inhibition of glycolysis induced by BRAFi/MEKi treatment [258].

Consistently with these observations, several *in vitro* or *in vivo* studies reproducing the induction of BRAFi or BRAFi/MEKi resistance successfully demonstrated an increase in mitochondrial respiration and subsequent ROS enhancement. In vemurafenib-resistant subclones of A375, SKMel28, and WM9 melanoma cells obtained both through *in vitro* treatment and *in vivo* treatment in xenografts in SCID mice, Corazao-Rosas and collaborators [259] demonstrated an increase in mitochondrial respiration and ROS production. Moreover, the elevated ROS level rendered vemurafenib-resistant melanoma cells both *in vitro* and *in vivo* more prone to cell death induced by a pro-oxidant drug, such as elesclomol. Similar results were obtained by Khamari and collaborators [260], who characterized the oxidative-stress-related metabolic adaptations in a preclinical murine model that accurately recapitulated *in vivo* the acquisition of resistance to BRAF or MEK inhibitors alone and in combination [260]. The resistance was obtained by treating SCID mice engrafted with A375 melanoma cells with vemurafenib. At first, the drug treatment drastically reduced tumors in all animals, but after a while, in some mice, melanoma cells were able to escape to anti-BRAF therapy and reinitiate growth. Then, melanoma cells were isolated from these relapsed mice. The vemurafenib-resistant A375 cell lines were also resistant to other BRAFi (dabrafenib) and MEKi (trametinib and cobimetinib) alone or in combination. As previously reported, these authors found out that these MAPK-resistant melanomas exhibited an enhancement of ROS production due to mitochondrial oxygen consumption increase. Interestingly, besides the enhancement of oxidative stress, they also reported an antioxidant adaptative response characterized by GSH accumulation. In particular, these authors showed that the increase in GSH was due to the increase in the availability of glutamate and cysteine, the two molecules that, together with glycine, form GSH. The cysteine increase can be ascribed to the observed upregulation of Nrf2, which in turn elicited an enhanced xCT expression, while the enhancement of glucose-derived glutamate was due to a reprogrammed mitochondrial metabolism [260].

ROS increase and oxidative DNA damage were observed in melanoma cellular models of double resistance to trametinib and dabrafenib both in *in vitro* and *in vivo*. Moreover, these double-resistant melanoma cells showed an increase in SOD2 levels. Inhibition of the antioxidant SOD2 or the use of an ROS scavenger such as NAC inhibited cellular growth in these MAPKi double-resistant cells [261].

### 4.1. Therapeutic Strategies to Overcome MAPKi Resistance

**Figure 3:**
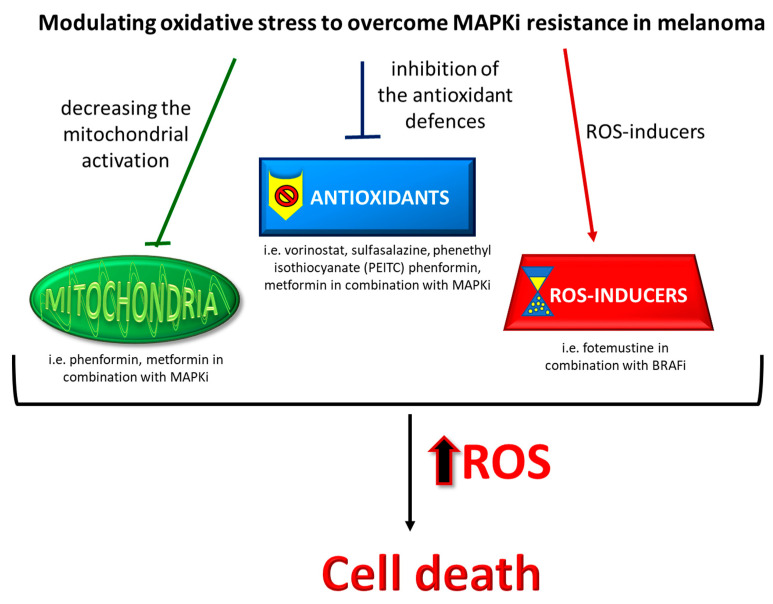
Modulating oxidative stress to overcome MAPKi resistance in melanoma. Three main therapeutical strategies able to modulate oxidative stress can be considered: (i) decreasing mitochondrial activation; (ii) inhibiting antioxidant defenses; (iii) further increasing ROS production. See Section 4.1 for full explanation.

To overcome BRAFi resistance in melanoma, three main therapeutical strategies able to modulate oxidative stress can be considered (Figure 3): (i) decrease in mitochondrial activation; (ii) inhibition of antioxidant defenses; and (iii) further increase in ROS production.

Targeting the metabolic rewiring from glycolysis toward OXPHOS represents an appealing therapeutical strategy in BRAFi/MEKi-resistant melanoma cells [127]. Several drugs have been used to target OXPHOS directly, such as phenformin and metformin, two biguanides used for treating type 2 diabetes; these two compounds enhanced the antitumor activities of BRAFi through the inhibition of the mitochondrial respiratory chain (MRC) complex I and the triggering of ROS production, which in combination with OXPHOS inhibition can be toxic for the cells [262,263]. Other inhibitors of the MRC complex I that can also induce ROS production, such as the small molecule inhibitor BAY 87-2243 and the plant extract deguelin, in association with the BRAFi vemurafenib can significantly reduce melanoma tumor growth when compared with their use as single agents [264,265,266,267].

Paralleling the increase in mitochondrial activation, which elicits higher ROS production, BRAFi/MEKi-resistant melanoma cells enhance their antioxidant systems to survive under oxidative stress. Therefore, targeting antioxidant defenses can be a therapeutical strategy to overcome BRAFi/MEKi resistance. Several shreds of evidence can sustain this hypothesis. Wang and collaborators demonstrated that the histone deacetylase inhibitor vorinostat also elicited a further, lethal increase in ROS by suppressing xCT expression [268]. Sulfasalazine, another xCT inhibitor, could delay the growth of BRAFi-resistant melanoma cells *in vitro* [260]. Phenethyl isothiocyanate (PEITC), an inhibitor of GST, could resensitize BRAFi-resistant melanoma cells to vemurafenib [269].

Finally, given the ROS overproduction in BRAFi/MEKi-resistant melanoma cells, another therapeutic strategy consists in further increasing ROS production with a pro-oxidant drug in combination with a MAPKi. This approach can then reconsider the old pro-oxidant chemotherapeutic drugs as an effective strategy to fight melanoma. In a recent phase 2 trial, BRAF V600 patients resistant to vemurafenib received a combined vemurafenib and fotemustine treatment that demonstrated clinical activity and an acceptable safety profile in BRAF-refractory patients [270].

## 5. Microbiota, Oxidative Stress, and Melanoma

In this complex scenario, the microbiota, the unique combination of microorganisms that is found within a specific environment, has emerged as another important protagonist. The gut microbiome (GM), the collection of genomes of all the microorganisms found in the gut, has been implicated in a wide range of human pathologies, including cancer [271]. Melanoma is not an exception. For instance, it was demonstrated that the intestinal microbial dysbiosis induced by antibiotics can increase the incidence of malignant melanoma in an animal model, and that treatment with probiotics, by restoring the microbiota diversity, significantly reduced tumor incidence [272]. Moreover, Jenkins and collaborators demonstrated that antibiotic-induced dysbiosis enhanced distal melanoma progression in B16-F10 tumor-bearing animals by altering the host cytokine profile; these changes were able in turn to inhibit the expression of vascular adhesion molecules and decrease the number of activated and effector CD8+ T-cells in tumors, highlighting the importance of commensal bacteria in supporting anticancer immune surveillance [273].

The GM has also been shown to affect response to melanoma therapies. Significant progress has been made by identifying the microbiome as a major player in the sensitivity to anti-PD1/PD-L1 and anti-CTLA4 immunotherapies in melanoma [274,275]. However, few studies have been conducted with the purpose of dissecting the role of microbiota in sensitivity to BRAFi/MEKi treatments or in modulating the onset of resistance to these targeted therapies. Among these few, it was demonstrated that the addition of inulin or mucin prebiotics to the diet of C57BL/6 mice induced different changes in gut microbiota taxa, followed by antitumor immune responses and inhibition of BRAF-mutant melanoma growth in a subcutaneously implanted syngeneic mouse model. Moreover, they observed that inulin, but not mucin, limited tumor growth in syngeneic mouse models of NRAS mutant melanoma and enhanced the efficacy of a MEKi with a delay in the development of drug resistance [276].

Interestingly, not only the GM but the intratumoral microbiome has gained attention in studies on microbiota’s impacts in response to several anticancer treatments (immunotherapy, chemotherapy, radiotherapy) [277,278]. In melanoma cells, the presence of *Fusobacterium nucleatum (F. nucleatum)*, a Gram-negative anaerobic periodontal pathogen involved in systemic diseases, inhibited the natural killer cytotoxicity towards tumor cells [279]; moreover, the highest amount of *F. nucleatum* in animal skins was found to be associated with a more aggressive melanoma disease [280].

It is well known that dysbiosis can be associated with oxidative stress; together with proinflammatory cytokines, it contributes to form a favorable *milieu* for the onset or the progression of several pathologies, such as metabolic syndrome, neurological disease, inflammatory bowel disease, and cancer [281]. Thus, in these conditions, innovative therapeutical strategies can be represented by the direct modulation of the microbiota, such as through the employment of polyphenolic nutraceuticals, to minimize oxidative stress and slow down inflammation [282]. In this regard, it has been shown that the administration of pomegranate aqueous extract, which is particularly rich in bioactive phytochemicals, inhibited tumor growth and showed with anti-inflammatory and antioxidant effects in a model of dimethylbenz(a)anthracene (DMBA)-initiated rat mammary tumorigenesis [283]. The connection among gut microbiota, oxidative, stress and melanoma is a largely unexplored area; however, some evidence has suggested their possible interplay. For instance, the genus *Lactobacillus,* with well-known anti-inflammatory and antioxidative effects [284], when administered in B16 melanoma-bearing animals, showed antimetastatic effects, reduced the incidence of melanoma and significantly prolonged survival [285,286,287].

## 6. Conclusions

There is no doubt that oxidative stress plays a role in melanoma initiation, progression, metastatic spread, and onset of chemoresistance to classical pro-oxidant drugs and MAPK-targeting therapies. However, controversial and opposite results on the role of ROS, LPO products, and their specific antioxidant or detoxifying systems in regulating cancer growth make it important to pay attention to the type of strategy to fight melanoma.

The decrease in ROS by using antioxidants may be detrimental in some cases, given the role of these substances in supporting tumor progression. On the contrary, inducing high ROS levels in melanoma cells, provided that high, lethal levels are surely reached, seems to have no contraindications. Probably the most promising strategies rely on the use of a combination of drugs that can affect multiple pathways at the same time.

Furthermore, new strategies of affecting redox balance by manipulating of the microbiota can be pursued.

A more complete understanding of the role of oxidative stress in melanoma disease will ensure the development of increasingly effective therapies.
